# Development of a Food Group-Based Diet Score and Its Association with Bone Mineral Density in the Elderly: The Rotterdam Study

**DOI:** 10.3390/nu7085317

**Published:** 2015-08-18

**Authors:** Ester A. L. de Jonge, Jessica C. Kiefte-de Jong, Lisette C. P. G. M. de Groot, Trudy Voortman, Josje D. Schoufour, M. Carola Zillikens, Albert Hofman, André G. Uitterlinden, Oscar H. Franco, Fernando Rivadeneira

**Affiliations:** 1 Department of Epidemiology, Erasmus MC, University Medical Centre, P.O. box 2040, 3000 CA Rotterdam, The Netherlands; E-Mails: e.a.l.dejonge@erasmusmc.nl (E.A.L.J.); trudy.voortman@erasmusmc.nl (T.V.); j.schoufour@erasmusmc.nl (J.D.S.); a.hofman@erasmusmc.nl (A.H.); a.g.uitterlinden@erasmusmc.nl (A.G.U.); o.franco@erasmusmc.nl (O.H.F.); 2 Department of Internal Medicine, Erasmus MC, University Medical Centre, P.O. box 2040, 3000 CA Rotterdam, The Netherlands; E-Mails: m.c.zillikens@erasmusmc.nl (M.C.Z.); f.rivadeneira@erasmusmc.nl (F.R.); 3 Department of Global Public Health, Leiden University College The Hague, P.O. box 13228, 2501 EE The Hague, The Netherlands; 4 Department of Human Nutrition, Wageningen University, P.O. box 8129, 6700 EV Wageningen, The Netherlands; E-Mail: Lisette.deGroot@wur.nl

**Keywords:** dietary patterns, bone mineral density, BMD-Diet score, healthy diet indicator

## Abstract

No diet score exists that summarizes the features of a diet that is optimal for bone mineral density (BMD) in the elderly. Our aims were (a) to develop a BMD-Diet Score reflecting a diet that may be beneficial for BMD based on the existing literature, and (b) to examine the association of the BMD-Diet Score and the Healthy Diet Indicator, a score based on guidelines of the World Health Organization, with BMD in Dutch elderly participating in a prospective cohort study, the Rotterdam Study (*n* = 5144). Baseline dietary intake, assessed using a food frequency questionnaire, was categorized into food groups. Food groups that were consistently associated with BMD in the literature were included in the BMD-Diet Score. BMD was measured repeatedly and was assessed using dual energy X-ray absorptiometry. The BMD-Diet Score considered intake of vegetables, fruits, fish, whole grains, legumes/beans and dairy products as “high-BMD” components and meat and confectionary as “low-BMD” components. After adjustment, the BMD-Diet Score was positively associated with BMD (β (95% confidence interval) = 0.009 (0.005, 0.012) g/cm^2^ per standard deviation). This effect size was approximately three times as large as has been observed for the Healthy Diet Indicator. The food groups included in our BMD-Diet Score could be considered in the development of future dietary guidelines for healthy ageing.

## 1. Introduction

Osteoporosis, characterized by low bone mineral density (BMD), is a major determinant of fracture risk and can lead to a decreased quality of life and loss of independency in the elderly [1]. An important and modifiable risk factor for osteoporosis is an inadequate diet [2]. Although studies on single nutrients, such as calcium and Vitamin D, have provided important insights on the relationship between diet and bone health [3], investigating full dietary patterns has additional benefits because additive or antagonistic nutrient-interactions might occur [4]. Two main approaches of dietary pattern identification can be distinguished. The first is an *a posteriori* approach, in which statistical data reduction techniques, such as factor or cluster analysis, are used to identify dietary patterns in a specific population [4]. This approach can be particularly useful to identify the local and existing dietary patterns as they are shaped by a variety of lifestyle factors, including individual preferences and beliefs, cultural traditions, and food availability and affordability [5]. Second, an *a priori* approach can be used, in which diet scores or diet indices are developed based on current knowledge from literature and guidelines.

Examples of diet scores are the Alternate Healthy Eating Index (AHEI) and the Recommended Food Score (RFS), which reflect diet quality based on the Dietary Guidelines for Americans and the food guide pyramid developed by researchers at the US Department of Agriculture. However, these scores were recently shown not to be associated with BMD in pre-menopausal women [6]. Accordingly, it may be argued that existing dietary scores based on existing dietary recommendations may not fully capture or consider foods that influence bone health.

Adherence to the dietary guidelines of the World Health Organization (WHO) [1] has been translated into the Healthy Diet Indicator (HDI) by Jankovic *et al.*, (2014) [7]. This score reflects the overall quality of a subject’s diet based on single nutrients (e.g., sodium) and some food groups (e.g., fruits and vegetables). The guidelines, and therefore the score, were developed based on existing evidence on dietary intake and chronic diseases, which included limited data from osteoporosis-related studies [1]. Moreover, as dietary guidelines are in transition to become food-group-based rather than nutrient-based, it would be valuable to develop a BMD-Diet Score based on the intake of food groups. By deriving these food groups from full dietary pattern analyses, this BMD-Diet Score might account for potential nutrient interactions. Eventually, it might serve the development of future food-group-based guidelines that sufficiently account for bone health.

In the present study, the first aim was to develop a BMD-Diet Score reflecting an overall diet that may be beneficial for BMD based on a narrative review of previously published *a priori* and *a posteriori* dietary pattern analyses on BMD. A second aim was to examine the association of the BMD-Diet Score and the Healthy Diet Indicator, a diet score based on current dietary guidelines of the WHO, with measured BMD and to compare these associations.

## 2. Experimental Section

### 2.1. Study Population

This study was embedded in the Rotterdam Study I (RS-I-1), a prospective cohort study among subject from the Ommoord district in Rotterdam, the Netherlands. Participants were elderly males and females of 55 years and older at baseline (1989–1993). Details on the design and main objectives of the Rotterdam Study have been published elsewhere [8]. The Rotterdam Study has been approved by the institutional review board (Medical Ethics Committee, MEC 89.230) of the Erasmus Medical Center and by the review board of The Netherlands Ministry of Health, Welfare and Sports [8]. Written informed consent was obtained from all subjects.

### 2.2. Dietary Assessment

All participants were interviewed at baseline for food intake assessment using an extensive semi-quantitative food frequency questionnaire (FFQ) at the study center, administered by a trained dietician. The questionnaire was validated and adapted for use in the elderly [9,10]. It consists of 170 food items and questions about dietary habits. The ability of the FFQ to rank subjects adequately according to their dietary intakes was demonstrated by results from a validation study comparing the FFQ to 15-day food records collected over a year to cover all seasons. Pearson’s correlation coefficients of this comparison ranged from 0.4 to 0.8 after adjustment for sex, age, total energy intake, and within-person variability in daily intakes [9]. The dietary intake of nutrients was calculated using the Dutch Food Composition Database (NEVO) from 1993 and 2006.

### 2.3. Development of the BMD-Diet Score

We searched PubMed for publications (through March 2015) on studies examining the relationship between dietary patterns and BMD using the following search terms: “Dietary patterns” OR “diet score” AND “bone” OR “BMD” OR “osteoporosis”. Studies included dietary patterns, derived by either cluster or factor analysis, or dietary indices as exposure and bone mineral density or loss thereof, or osteoporosis as outcome in adult populations. Selected studies on single food groups, single dietary nutrients or nutrient biomarkers as exposure and outcomes other than bone mineral density or osteoporosis were excluded. Furthermore, we excluded specific diseased populations, such as celiac disease patients and studies in children (because their dietary patterns may differ of those from adults and they are still undergoing bone accrual). We only considered original research (observational and experimental) and no case reports.

We extracted food groups from dietary patterns and labelled them as “high-BMD” or “low-BMD” food groups if significant associations (*p* < 0.05) were reported with high or low BMD, respectively. Characterization as well as labelling of dietary patterns derived by principal component analysis was based on their factor loadings, which represent the correlation between the food groups and the dietary patterns. However, different studies might use different factor loading- thresholds. Because not all studies reported smaller factor loadings, we only included food groups with a factor loading of >0.3 for positively correlated food groups and <−0.3 for negatively correlated food groups. Next, we created bar charts presenting the count of dietary patterns in which any of these food groups occurred. Only those with the highest frequency of occurrence (>25th percentile of cumulative count) were included for the BMD-Diet Score. The direction of the association (favorable or unfavorable) was considered consistent when more than two thirds (67%) of the studies showed an effect in the same direction. Only food groups with consistent associations with BMD were included in the BMD-Diet Score.

For each participant, the newly developed BMD-Diet Score was calculated as follows: first, dietary intake of all relevant food groups was categorized into quartiles. Next, each subject was assigned ascending values (1,2,3,4) for food groups that are assumed to increase BMD and descending values (4,3,2,1) for those assumed to decrease BMD, based on their quartiles of intakes. Only if the distribution of intake of a food group did not allow computation of quartiles (e.g., for groups with a high number of non-consumers such as legumes and beans), values were dichotomized. Intake of alcoholic beverages was not included in the BMD-Diet Score but considered a potential confounder in our analysis, because the relationship with BMD might be non-linear [11,12].

### 2.4. Computation of HDI, Based on Dietary Guidelines of the WHO

The computation of the HDI for each participant was based on WHO dietary guidelines of 2003. Briefly, the HDI consists of 12 dietary components, of which 5 are recommended to be consumed in moderation: saturated fatty acids (SFA), mono-and disaccharides, cholesterol, trans fat and sodium, three components which are recommened to consume within a specific range: polyunsaturated fatty acids (PUFAs), protein, total fat, *n*-6 PUFAs and *n*-3 PUFAs, and two components for which an adequate intake is recommended: dietary fiber and fruits and vegetables. Cut-offs and more detailed information regarding the scoring system are presented in Table 1. The HDI is coded as a continuous variable, proportionally ranging from 10 to 0 between the optimal intake and the lower or upper limit respectively per component. Therefore, the theoretical range of HDI is 0 to 120.

**Table 1:** Cut-offs used for computation of the Healthy Diet Indicator (HDI) (Jankovic, 2014 [7], adapted).

Components of the Healthy Diet Indicator	Lower Limit	Optimal Intake *	Upper Limit **
	0 Points	10 Points	0 Points
**Moderation (unfavorable) components**			
Saturated fatty acids	N.A.	<10	>15
Monosaccharides and disaccharides	N.A.	<10	>30
Cholesterol	N.A.	<300	>400
Trans fatty acids	N.A.	<1	>1.5
Sodium (grams, not sodium chloride)	N.A.	<2	>3.0
**Moderation range components**			
Polyunsaturated fatty acids (PUFAs)	0	6 to 10	>10
Protein	0	10 to 15	>20
Total fat	0	15 to 30	> 43
*n*-6 PUFA	0	5 to 8	>8.5
*n*-3 PUFA	0	1 to 2	N.A. **
**Adequacy (favorable) components**			
Dietary fiber (g)	0	>25	N.A.
Fruits and vegetables (g)	0	>400	N.A.

*: Representing the World Health Orgnization (WHO) recommendation; ** For *n*-3 PUFA’s no upper level could be calculated as the 85th percentile of intake falls within the range of optimal intake in our population; Abbreviations: N.A. = not applicable, PUFA = polyunsaturated fatty acid; The Healthy Diet Indicator (HDI) is coded as a continuous variable, proportionally ranging from 10 to 0 between the optimal intake and the lower or upper limit respectively.

### 2.5. Assessment of BMD

BMD of the femoral neck was measured by dual energy X-ray absorptiometry (DXA) using a Lunar DPX- densitometer (Lunar Radiation Corp., Madison, WI, USA) at baseline (1989–1993) and at 3 subsequent visits (1993–1995, 1997–1999 and 2002–2004). DXA scans were analyzed with DPX-IQ software (v.4.7d) and BMD values are expressed in g/cm^2^. A flowchart showing the numbers of subjects with available BMD data for each visit is shown in Figure S1.

### 2.6. Assessment of Covariates

We included covariates related to body composition, lifestyle, socioeconomic status (SES), prevalent metabolic diseases, use of medication and other indicators of overall health, of which the majority was assessed at baseline (1989–1993). Body height and weight were measured at the research center at baseline and three follow up visits (1993–1995, 1997–1999 and 2002–2004). Regarding lifestyle factors, smoking at baseline was calculated as “current” or “past or never”. Physical activity was assessed at the 3rd visit (1997–1999), using the Zutphen Study Physical Activity Questionnaire including questions on walking, cycling, gardening, diverse sports, hobbies, and housekeeping [13,14,15]. Total time spend on physical activity was calculated by the sum of minutes per week for each type of activity. Dietary intake of alcoholic beverages and calcium were derived from the FFQ. Baseline use of any dietary supplement was assessed during the home interview, without specific questions on dose or duration and coded as “never” or “ever”. Highest education and net household income were used as proxy for SES. Education was coded as “low” (primary education, primary + higher not completed, lower vocational and lower secondary education) or “high” (intermediate vocational, general secondary, higher vocational education and university). Household income was coded “above” or “below” the average of 2400 net Dutch Guilders (≈1600 Euro) per month. Regarding prevalent diseases at baseline, type 2 diabetes mellitus was determined as baseline serum glucose concentrations >11 mmol/L or use of glucose lowering drugs and cardiovascular disease included prevalent coronary heart disease, heart failure, stroke and arterial fibrillation at baseline. Methods of data collection and definitions of cardiac outcomes in the Rotterdam Study have been described in detail elsewhere [16]. Regarding medication, the use of serum lipid reducing agents, antihypertensive drugs, or drugs taken for calcium homeostasis and disorders of the musculoskeletal system was registered during the home interview by trained research assistants [17]. Use of hormone replacement therapy (HRT) in females was coded as “never” or “ever”. Lower limb disability and Vitamin D status were included as remaining measures of overall health. Lower limb disability index, a combined index reflecting a subject’s ability to stand up, walk, climb and bend [18] was based on the Stanford Health Assessment Questionnaire. Serum 25-hydroxyvitamin D (25(OH)D) was measured in a subgroup of participants (*n* = 3171) at the 3rd visit of the cohort to the visiting center using radioimmunoassays (IDS Ltd, Boldon, UK). The sensitivity of the test was 3 nmol/L which ranged from 4 to 400 nmol/L. Intra-assay accuracy was <8% and the inter-assay accuracy was <12%.

### 2.7. Statistical Analysis

Characteristics of the study population were provided for subjects with a BMD Diet-Score below or above the median separately. Median values (+ interquartile ranges) for continuous variables and percentages of the total population for categorical variables were provided. The association between the BMD-Diet Score and HDI with BMD was studied using linear mixed modelling with the diet scores, expressed in standard deviations (SDs) or in quartiles, as exposure and longitudinal measurements of BMD (expressed in g/cm^2^ and sex-specific *z*-scores) as the outcome. Analyses in quartiles, using the lowest quartile as the reference category, were performed to explore potential non- linear relationships. We coded the time- variable in the mixed model 0, 2, 6.5 and 11, to correct for differences in the length of time- intervals between subjects. Basic models (model 1) were adjusted for age and sex only. Potential confounding was tested by adding covariates to the models separately. Only covariates that changed the effect estimates by >10% were kept in the final adjusted models [19]. Based on this criterion, analyses were adjusted for age, sex and total kilocalorie intake, plus body weight and height (model 2), education, household income, current smoking behavior, physical activity, prevalent type 2 diabetes at baseline, and use of lipid lowering drugs, alcohol consumption and dietary supplement use (model 3). To assess whether BMD Diet-score had additional value upon the HDI, we further adjusted the final model for the HDI diet score (model 4). The aim of this paper is to study associations between diet scores reflecting full dietary patterns, not single nutrients, in relation to BMD. However, as the nutrient calcium is one of the most important constituents of the bone, we investigated the effects of additional adjustment for calcium intake in a separate model (model 5). To be able to study whether the trajectories of BMD were different in subjects with low or high diet scores, we tested for interaction with time by adding the product term of BMD-Diet Score or HDI with time to model 3.

We used a multiple imputation procedure to estimate missing values for covariates (details in Tables S1 and S2). To facilitate proper comparison of the effect estimates of associations between the BMD-Diet Score (ranging from 0 to 30) with BMD with that of the HDI (ranging from 0 to 120) with BMD, the regression coefficients were shown per SD increase for both diet scores.

As the majority of studies that served as a basis for our BMD-Diet Score were performed in women only, we tested for interaction with sex, by adding the product term of the our main exposures (the two diet scores) and sex to our basic models. Additionally, we performed a sensitivity analysis excluding participants with type 2 diabetes at baseline. All analyses were performed using SPSS 22 (IBM, Chicago, IL, USA) and R 3.1.2 (The R Foundation for Statistical Computing, Vienna, Austria) statistical software.

## 3. Results

### 3.1. Food Groups Included in Our BMD-Diet Score

In summary, we identified 15 papers to be used for the development of our BMD-Diet Score. The majority of these studies investigated *a posteriori* defined dietary patterns using principal component analysis [20,21,22,23,24,25,26,27,28,29,30,31] or cluster analysis [32]. Details on these studies regarding their design, sample size and food group extracted are shown in Table S3 and S4. Studies on *a priori* defined diet scores and BMD showed positive effects for the Mediterranean Diet Score [33], the Dietary Diversity Score [23,34] and the Diet and Lifestyle Score, based on guidelines of the American Heart Association [35] (Table S5).

**Figure 1:**
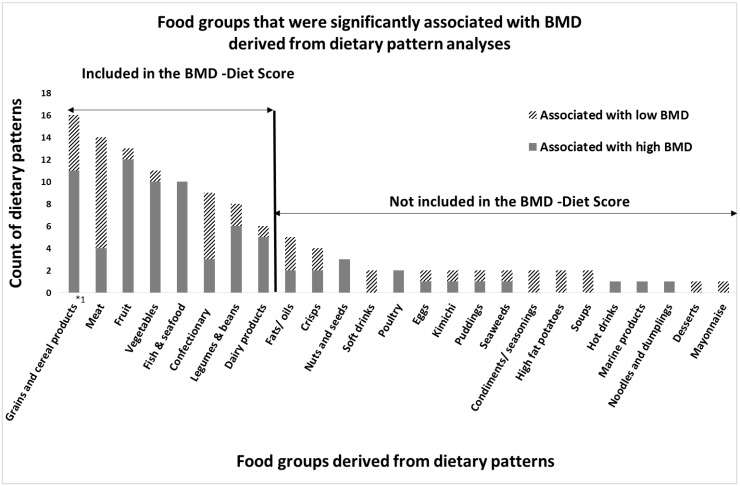
Results of the narrative review: Food groups that were associated with high or low bone mineral density (BMD) in dietary pattern analyses; The X-axis displays the food groups, derived from dietary patterns that were significantly associated with high or low BMD in the reviewed literature. The Y-axis displays the number of dietary patterns in which corresponding food group occurred (count of dietary patterns). As some studies report more than one dietary pattern to be associated with BMD, the number of patterns that was counted is slightly different from the number of studies that was counted. *1: Although not all studies distinguished between refined and whole grains, those that did found particularly beneficial associations with bone for whole grains only.

After careful evaluation of the available evidence, eight food groups were included in the BMD Diet-score: vegetables, fruits, dairy products, whole grain products, fish and legumes & beans as “High-BMD” components and meat (including red, processed and organ meat) and confectionary (including candies, cakes and cookies) as “Low-BMD” components (Figure 1). An overview of food items included in each food group is shown in Table S6.

### 3.2. Characteristic of the Study Population

Characteristics of the study population are shown in Table 2. Subjects with a BMD-Diet Score above the median were more likely to be female (62% *vs*. 56%) and to have a higher income (54% *vs*. 49%) than those with a BMD-Diet Score below the median. Furthermore, they were less likely to be smokers (27% *vs*. 19%) and had higher calcium intakes (median of 1248 mg/day *vs.* 960 mg/day).

**Table 2:** Characteristics of the study population in participants with a BMD-Diet Score below or above the median.

	BMD-Diet Score below or Equal to the Median ^2^	BMD-Diet Score above the Median	Total
*n*	2903	2241	5144
Age (year) ^1^	68 (61, 73)	65 (60, 71)	67 (61, 73)
Total energy intake (kcal/day) ^1^	1926 (1613, 2265)	1921 (1617, 2254)	1923 (1615, 2261)
Dietary calcium intake (mg/day) ^1^	960 (769, 1170)	1248 (1032, 1497)	1079 (863, 1324)
Physical activity (h/day)	5.6 (4.0, 7.5)	6.0 (4.4, 8.0)	5.8 (4.2, 7.7)
Of which vigorous (h/day) ^1^	0.4 (0.1, 0.9)	0.6 (0.2, 1.1)	0.5 (0.2, 1.0)
Height (cm) ^1^	167 (161, 174)	167 (160, 174)	162(157,166)
Weight (kg) ^1^	73 (65, 80)	74 (66, 81)	73 (66, 81)
Healthy Diet Indicator ^1^	74 (66, 82)	79 (70, 86)	76 (68, 84)
Plasma Vitamin D (nmol/L) ^1,3^	44 (29, 64)	45 (31, 65)	45 (30, 64)
Sex (% females)	56	62	57
Prevalent osteoporosis (%)	12	10	11
Prevalent type 2 diabetes (%)	9	10	10
Prevalent cardiovascular disease (%)	13	12	13
High education (%)	35	39	37
Monthly income > 1600 Euro (%)	49	54	51
Current smokers (%)	27	19	23
Current or past HRT use (%) ^4^	8	11	9
Lipid lowering drug use (%)	2	3	3
Antihypertensive drug use (%)	13	13	13
Lower limb disabled (%)	19	16	17

^1^: median (interquartile range); ^2^: the median of the BMD-Diet Score in our population is 19; ^3^: assessed at the 3rd visit; ^4^: expressed as percentages of the female population; Abbreviations: BMD = Bone mineral density; HRT = hormone replacement therapy.

The BMD-Diet Score and the HDI were weakly but significantly correlated (Pearson’s ρ = 0.18). The HDI, but not the BMD-Diet Score, was significantly correlated with lower total energy intake (Pearson’s ρ = −0.23). Median intake of each food group included in the BMD-Diet Score is shown in Table S7.

### 3.3. Longitudinal Associations between BMD-Diet Score, HDI and BMD

Associations between the BMD-Diet Score or the HDI and BMD, are shown in Table 3. Adjusted for age, sex and total energy intake (model 1), a high BMD-Diet Score was significantly associated with higher BMD (β (95% confidence interval (CI)) = 0.012 (0.008, 0.015) g/cm^2^ per SD increase in the diet score). This association was slightly attenuated (β (95% CI) = 0.010 (0.007, 0.013) after adjustment for body height and weight (model 2) and after including additional confounders (β (95% CI) = 0.009 (0.005, 0.012), model 3). Additional adjustment for adherence to the HDI did not change the results (model 4). After further adjustment for dietary calcium intake effect sizes were diluted, but remained significant (β (95% CI) = 0.004 (0.001, 0.009) g/cm^2^ per SD increase in the diet score). No significant interaction with time was observed (*p* for interaction = 0.25), indicating that the trajectories of BMD were comparable between subjects with high or low BMD-Diet Scores.

The HDI was significantly associated with higher BMD in the basic model. However, after adjustment for age, sex, height and weight (model 2) the standardized effect size decreased (β (95% CI) = 0.005 (0.002, 0.008) and was of a lesser magnitude than that of the BMD-Diet Score. After adjustment for confounders the association was diluted and became non-significant (β (95% CI) = 0.003 (−0.000, 0.007) in model 3). Further adjustment for adherence to the BMD-Diet Score did not change this effect (model 4), while a positive association was observed after additional adjustment for calcium intake (model 5). No significant interaction with time was observed (*p* for interaction = 0.18).

Categorical analyses, using the lowest quartile as the reference group, did not indicate the presence of a non- linear relationship between the BMD- Diet Score or the HDI with BMD (Table 3).

### 3.4. Additional Analysis

No interaction between the BMD-Diet Score or the HDI with sex was observed in relation to BMD (*p* all interactions > 0.12). Additionally, stratification by gender did not show different associations for males and females. Additional analyses with BMD in sex- specific *z*-scores as the outcome did not change the results. In addition, sensitivity analyses in which participants were excluded if they had type 2 diabetes at baseline did not change the results.

**Table 3:** Associations between the BMD-Diet Score or Healthy Diet Indicator and femoral neck BMD, using linear mixed modelling.

		Model 1		Model 2		Model 3		Model 4		Model 5	
		*Basic*		*Model 1+*		*Model 2+*		*Model 3+*		*Model 3+*	
				*Height and Weight*		*Confounders*		*Other Score*		*Calcium Intake*	
		β ^1^	95% CI	β	95% CI	β	95% CI	β	95% CI	β	95% CI
**Food group-based BMD-Diet Score**	**Per SD**	**0.012**	**(0.008, 0.015)**	**0.010**	**(0.007, 0.013)**	**0.009**	**(0.005, 0.012)**	**0.008**	**(0.004, 0.011)**	**0.004**	**(0.001, 0.008)**
	**Q2 *vs.* Q1**	0.007	(–0.004, 0.018)	0.007	(–0.003, 0.016)	0.005	(–0.004, 0.015)	0.005	(–0.004, 0.015)	0.001	(–0.009, 0.012)
	**Q3 *vs.* Q1**	**0.024**	**(0.014, 0.034)**	**0.020**	**(0.011, 0.030)**	**0.019**	**(0.009, 0.028)**	**0.018**	**(0.008, 0.028)**	**0.019**	**(0.002, 0.022)**
	**Q4 *vs.* Q1**	**0.029**	**(0.020, 0.040)**	**0.024**	**(0.016, 0.033)**	**0.022**	**(0.013, 0.031)**	**0.021**	**(0.012, 0.030)**	**0.010**	**(0.000, 0.020)**
	***P* for trend**	**<0.001**		**<0.001**		**<0.001**		**<0.001**		**0.016**	
**WHO guidelines-based HDI**	**Per SD**	**0.004**	**(0.001, 0.008)**	**0.005**	**(0.002, 0.009)**	0.003	(−0.000, 0.007)	0.003	(−0.000, 0.007)	**0.005**	**(0.003, 0.010)**
	**Q2 *vs.* Q1**	–0.006	(–0.017, 0.005)	–0.006	(–0.016, 0.005)	–0.008	(–0.018, 0.003)	–0.009	(–0.020, 0.002)	–0.007	(–0.018, 0.003)
	**Q3 *vs.* Q1**	**0.006**	(–0.026, 0.016)	**0.007**	(–0.012, 0.016)	0.004	(–0.004, 0.013)	0.002	(–0.007, 0.010)	0.005	(–0.004, 0.013)
	**Q4 *vs.* Q1**	**0.011**	(–0.000, 0.021)	**0.012**	**(0.002, 0.022)**	0.007	(–0.004, 0.017)	0.002	(–0.009, 0.012)	0.008	(–0.002, 0.018)
	***P* for trend**	**0.014**		**0.003**		0.067		0.377		**0.038**	

^1^ Regression coefficients (+95% confidence intervals) are shown for the fixed effects of the linear mixed model per SD increase or per quartile, using the first quartile as the reference, in the corresponding diet score. As the median BMD in this population is 0.86 g/cm^2^, a regression coefficient of 0.012 g/cm^2^ approximates a 1.4% higher BMD; Model 1: Adjusted for age, sex and total energy intake; Model 2: Model 1, additionally adjusted for body weight and height; Model 3: Model 2, additionally adjusted for education, household income, smoking behavior, physical activity, use of lipid lowering drugs + use of any dietary supplement + alcohol intake. *Additional adjustment for plasma vitamin D*, *use of antihypertensive drugs, drugs for calcium homeostasis or for disorders of the musculo-skeletal system*, *HRT*, *lower limb disability or CVD prevalence did not change these results*; Model 4: Model 3, additionally adjusted for the other diet Score (HDI for the BMD-Diet Score analysis and vice versa); Model 5: Model 3, additionally adjusted for calcium intake; Significant findings (*p* < 0.05) in **bold**, Abbreviations: BMD = bone mineral density, HDI = Healthy Diet Indicator, HRT = hormone replacement therapy, CVD = cardiovascular disease, SD = standard deviation; CI = Confidence interval; Q = quartile.

## 4. Discussion

### 4.1. Summary of Main Findings

This is the first study in which a food group-based BMD-Diet Score based on existing evidence from previous studies on full dietary patterns and BMD in several populations has been developed. We found that this newly developed BMD-Diet Score was significantly associated with high BMD, independent of adherence to the dietary recommendations of the WHO as assessed by the HDI. Our findings suggest that there is room for improvement of current dietary guidelines seeking optimal bone health.

### 4.2. Comparison to Existing Scores That were Shown to Favorably Affect Markers of Bone Turnover

Our BMD-Diet Score was developed based on studies investigating the effects of dietary patterns on BMD. However, the associations between existing diet scores have also been studied in relation to other bone-related outcomes, such as markers of bone turnover. For example, the “Dietary Approaches to Stop Hypertension” (DASH)-Diet score was shown to favorably affect osteocalcin, a serum marker of bone formation, which, if sustained, may improve bone mineral status [36] and reduce bone loss.

The DASH-Diet score and our BMD-Diet Score share common components, namely fruits, vegetables, fish, and whole grains as favorable (high-BMD) food groups and (red) meat as unfavorable (low-BMD) food groups. However, the DASH-Diet score does include dairy products as favorable components, similar to our BMD-Diet Score, but uses a more specific definition by including only low fat dairy products [36]. Additionally, the study by Karamati *et al*., (2012) [28] showed a dietary pattern including low fat dairy to be associated with high BMD, and a pattern including high fat dairy to be associated with low BMD. Based on these findings, it could be argued that the BMD-Diet Score might be refined further by using low fat instead of all dairy products as a favorable component. The DASH-Diet score includes total fat as unfavorable nutrient-component (Table S8). Our BMD-Diet Score was based solely on food groups and therefore has no specific fatty acid-component. However, it includes foods as pork, cake, and chocolate bars, products high in saturated fatty acids, in the unfavorable “low BMD” components (Table S6), and fish, rich in polyunsaturated fatty acids, as favorable component. Therefore, our BMD-Diet Score could be considered a score in which the existing DASH-diet score was covered, but was fully translated into food groups.

### 4.3. Potential Nutrients Involved

The aim of this paper is to study the associations between complete dietary patterns, reflected by different diet scores in relation to BMD. However, calcium is a vital element of bone and a well-established dietary factor that influences BMD [3,37,38]. Our analysis showed the associations between the BMD-Diet Score and BMD were diluted, but remained significant after additional adjustment for dietary calcium. This indicates that calcium intake is important, but does not fully explain the favorable association between the BMD-Diet Score and BMD. This finding is in line with an earlier review by Ahmadieh *et al.*, (2001) who highlighted the positive contributions of a variety of nutrients to BMD, such as Vitamin B2, B6, Vitamin C and Vitamin K, in addition to calcium [2]. These nutrients can underlie our associations, since vitamin B2 and B6 might be reflected by the whole grain component of our BMD-diet Score and Vitamin C and K1 by the fruits and vegetable components.

### 4.4. Strengths and Limitations

Our study has several strengths. Firstly, the development of our BMD-Diet Score was based on a variety of study populations, including both Caucasian and Asian subjects. Despite the differences in dietary habits between these populations, we were able to identify common food groups that were consumed and were shown to be associated with BMD across populations. Secondly, by using full dietary pattern analyses as a basis for the BMD-Diet Score, we were able to take into account strong correlations and potential interactions between foods and nutrients. Thirdly, we had the opportunity to include repeated measurements of BMD, body weight, and height. Repeated BMD measurements provided more insights into long-term associations between dietary intake and BMD and the opportunity to study associations with BMD trajectories. Repeated measurements of body weight and height enabled a precise adjustment for changes in anthropometric measures, which are known to be important determinants of BMD. Lastly, our sample included both males and females, increasing the external validity of our results since most studies on dietary patterns and BMD focused on women only.

We do, however, also recognize some limitations. Our study population consisted of Dutch participants from one specific neighborhood, in which the vast majority of inhabitants were of Caucasian background, an aspect that is important to consider when extrapolating our findings to other populations. The absolute intakes of some components of the BMD-Diet Score (such as fish and legumes) were very low in our population, which might have affected the strength of our associations. However, for the main food groups, including fruits, vegetables, fish and whole grain products, we believe this concern is limited since items in these food groups are widely consumed in our population. It could be argued that using results from Rotterdam Study for the development of the BMD-Diet Score while subsequently testing the association between this score with BMD in the same cohort might have led to bias. However, the composition of the BMD-Diet Score would be similar with or without inclusion of our own previous results [24] (Tables S3 and S4) in its development. Therefore, we believe that inclusion of our previous results did not lead to bias in this study.

### 4.5. Future Steps and Implications

This is the first study that developed a BMD Diet Score that has been associated with BMD in a Dutch population of elderly subjects. Although the score is based on data from different populations, it is essential to study its performance in other populations, including Asian and other non-Caucasian populations. For example populations with (a) low dairy intake or (b) higher levels of Vitamin D or (c) high intake of foods that were hardly consumed in our population such as fish or legumes would be particularly interesting for replication. If future studies replicate positive associations with BMD, this BMD-Diet Score could help to shape food group-based dietary guidelines aiming to contribute to healthy ageing while considering a healthy BMD as important aspect of ageing. However, since dietary guidelines aim to promote overall healthy ageing by preventing all chronic diseases such as cardiometabolic diseases and cancer, our BMD-Diet Score should be studied in relation to these health outcomes as well. Calcium might favor BMD while adversely affecting cardiovascular disease risk [39], whereas an approach which evaluates the full diet, such as the BMD-Diet Score, might indicate benefit for various aspects of healthy ageing simultaneously.

For the development of our BMD-Diet Score we only used studies with BMD, and not fracture risk, as the primary outcome. However, as adherence to the Mediterranean Diet Score, for example, has been shown to be favorably associated with fracture risk in a cohort of adults from eight European countries [40], consumption of the food groups in our proposed BMD-Diet Score might favorably affect fracture risk as well.

## 5. Conclusions

We developed a new BMD-Diet Score composed of components representing high intake of vegetables, fruits, fish, whole grains, legumes and beans, and dairy products, and low intake of and meat and confectionary. This BMD-Diet Score is positively associated with BMD in our cohort of middle-aged and elderly subjects independent of adherence to the HDI based on dietary guidelines from the WHO. The food groups included in our BMD-Diet Score could be considered in the development of future dietary guidelines for healthy ageing.

## Supplementary Files

Supplementary File 1

## References

[1] World Health Organisation World Health Organisation Scientific Group on the Assessment of Osteoporosis at Primary Health Care Level. Summary Meeting Report.

[2] Ahmadieh H., Arabi A. (2011). Vitamins and bone health: Beyond calcium and Vitamin D. Nutr. Rev..

[3] Zhu K., Prince R.L. (2012). Calcium and bone. Clin. Biochem..

[4] Hu F.B. (2002). Dietary pattern analysis: A new direction in nutritional epidemiology. Curr. Opin. Lipidol..

[5] Ocke M.C. (2013). Evaluation of methodologies for assessing the overall diet: Dietary quality scores and dietary pattern analysis. Proc. Nutr. Soc..

[6] Zagarins S.E., Ronnenberg A.G., Gehlbach S.H., Lin R., Bertone-Johnson E.R. (2012). Are existing measures of overall diet quality associated with peak bone mass in young premenopausal women?. J. Hum. Nutr. Diet..

[7] Jankovic N., Geelen A., Streppel M.T., de Groot L.C., Orfanos P., van den Hooven E.H., Pikhart H., Boffetta P., Trichopoulou A., Bobak M. (2014). Adherence to a healthy diet according to the world health organization guidelines and all-cause mortality in elderly adults from Europe and the United States. Am. J. Epidemiol..

[8] Hofman A., Murad S.D., van Duijn C.M., Franco O.H., Goedegebure A., Ikram M.A., Klaver C.C.W., Nijsten T.E.C., Peeters R.P., Stricker B.H.C. (2013). The Rotterdam Study: 2014 objectives and design update. Eur. J. Epidemiol..

[9] Klipstein-Grobusch K., den Breeijen J.H., Goldbohm R.A., Geleijnse J.M., Hofman A., Grobbee D.E., Witteman J.C.M. (1998). Dietary assessment in the elderly: Validation of a semiquantitative food frequency questionnaire. Eur. J. Clin. Nutr..

[10] Goldbohm R.A., van den Brandt P.A., Brants H.A., van’t Veer P., Al M., Sturmans F., Hermus R.J. (1994). Validation of a dietary questionnaire used in a large-scale prospective cohort study on diet and cancer. Eur. J. Clin. Nutr..

[11] Sommer I., Erkkila A.T., Jarvinen R., Mursu J., Sirola J., Jurvelin J.S., Kroger H., Tuppurainen M. (2013). Alcohol consumption and bone mineral density in elderly women. Public Health Nutr..

[12] Rapuri P.B., Gallagher J.C., Balhorn K.E., Ryschon K.L. (2000). Alcohol intake and bone metabolism in elderly women. Am. J. Clin. Nutr..

[13] Caspersen C.J., Bloemberg B.P., Saris W.H., Merritt R.K., Kromhout D. (1991). The prevalence of selected physical activities and their relation with coronary heart disease risk factors in elderly men: The Zutphen Study, 1985. Am. J. Epidemiol..

[14] Stel V.S., Smit J.H., Pluijm S.M., Visser M., Deeg D.J., Lips P. (2004). Comparison of the LASA physical activity questionnaire with a 7-day diary and pedometer. J. Clin. Epidemiol..

[15] Voorrips L.E., Ravelli A.C., Dongelmans P.C., Deurenberg P., Van Staveren W.A. (1991). A physical activity questionnaire for the elderly. Med. Sci. Sports Exerc..

[16] Leening M.J.G., Kavousi M., Heeringa J., van Rooij F.J.A., Verkroost-van Heemst J., Deckers J.W., Mattace-Raso F.U.S., Ziere G., Hofman A., Stricker B.H.C. (2012). Methods of data collection and definitions of cardiac outcomes in the Rotterdam Study. Eur. J. Epidemiol..

[17] Sjahid S.I., van der Linden P.D., Stricker B.H. (1998). Agreement between the pharmacy medication history and patient interview for cardiovascular drugs: The Rotterdam Elderly Study. Br. J. Clin. Pharmacol..

[18] Burger H., de Laet C.E.D.H., van Daele P.L.A., Weel A.E.A.M., Witteman J.C.M., Hofman A., Pols H.A.P. (1998). Risk factors for increased bone loss in an elderly population—The Rotterdam Study. Am. J. Epidemiol..

[19] Mickey R.M., Greenland S. (1989). The impact of confounder selection criteria on effect estimation. Am. J. Epidemiol..

[20] Shin S., Joung H. (2013). A dairy and fruit dietary pattern is associated with a reduced likelihood of osteoporosis in Korean postmenopausal women. Br. J. Nutr..

[21] Shin S., Sung J., Joung H. (2014). A fruit, milk and whole grain dietary pattern is positively associated with bone mineral density in Korean healthy adults. Eur. J. Clin. Nutr..

[22] Mu M., Wang S.F., Sheng J., Zhao Y., Wang G.X., Liu K.Y., Hu C.L., Tao F.B., Wang H.L. (2014). Dietary patterns are associated with body mass index and bone mineral density in Chinese freshmen. J. Am. Coll. Nutr..

[23] Whittle C.R., Woodside J.V., Cardwell C.R., McCourt H.J., Young I.S., Murray L.J., Boreham C.A., Gallagher A.M., Neville C.E., McKinley M.C. (2012). Dietary patterns and bone mineral status in young adults: The Northern Ireland young hearts project. Br. J. Nutr..

[24] De Jonge E.A.L., Rivadeneira F., Erler N.S., Hofman A., Uitterlinden A.G., Franco O.H., Kiefte-de Jong J.C. (2015). Dietary patterns in an elderly population and their relation with bone mineral density: The Rotterdam Study.

[25] Fairweather-Tait S.J., Skinner J., Guile G.R., Cassidy A., Spector T.D., MacGregor A.J. (2011). Diet and bone mineral density study in postmenopausal women from the twinsuk registry shows a negative association with a traditional English dietary pattern and a positive association with wine. Am. J. Clin. Nutr..

[26] Hardcastle A.C., Aucott L., Fraser W.D., Reid D.M., Macdonald H.M. (2011). Dietary patterns, bone resorption and bone mineral density in early post-menopausal Scottish women. Eur. J. Clin. Nutr..

[27] McNaughton S.A., Wattanapenpaiboon N., Wark J.D., Nowson C.A. (2011). An energy-dense, nutrient-poor dietary pattern is inversely associated with bone health in women. J. Nutr..

[28] Karamati M., Jessri M., Shariati-Bafghi S.E., Rashidkhani B. (2012). Dietary patterns in relation to bone mineral density among menopausal Iranian women. Calcif. Tissue Intl..

[29] Langsetmo L., Poliquin S., Hanley D.A., Prior J.C., Barr S., Anastassiades T., Towheed T., Goltzman D., Kreiger N. (2010). Dietary patterns in Canadian men and women ages 25 and older: Relationship to demographics, body mass index, and bone mineral density. BMC Musculoskelet. Disord..

[30] Kontogianni M.D., Melistas L., Yannakoulia M., Malagaris I., Panagiotakos D.B., Yiannakouris N. (2009). Association between dietary patterns and indices of bone mass in a sample of Mediterranean women. Nutrition.

[31] Okubo H., Sasaki S., Horiguchi H., Oguma E., Miyamoto K., Hosoi Y., Kim M.K., Kayama F. (2006). Dietary patterns associated with bone mineral density in premenopausal Japanese farmwomen. Am. J. Clin. Nutr..

[32] Tucker K.L., Chen H., Hannan M.T., Cupples L.A., Wilson P.W., Felson D., Kiel D.P. (2002). Bone mineral density and dietary patterns in older adults: The Framingham Osteoporosis Study. Am. J. Clin. Nutr..

[33] Rivas A., Romero A., Mariscal-Arcas M., Monteagudo C., Feriche B., Lorenzo M.L., Olea F. (2013). Mediterranean diet and bone mineral density in two age groups of women. Intl. J. Food Sci. Nutr..

[34] Go G., Tserendejid Z., Lim Y., Jung S., Min Y., Park H. (2014). The association of dietary quality and food group intake patterns with bone health status among Korean postmenopausal women: A study using the 2010 Korean national health and nutrition examination survey data. Nutr. Res. Pract..

[35] Bhupathiraju S.N., Lichtenstein A.H., Dawson-Hughes B., Hannan M.T., Tucker K.L. (2013). Adherence to the 2006 American heart association diet and lifestyle recommendations for cardiovascular disease risk reduction is associated with bone health in older Puerto Ricans. Am. J. Clin. Nutr..

[36] Lin P.H., Ginty F., Appel L.J., Aickin M., Bohannon A., Garnero P., Barclay D., Svetkey L.P. (2003). The Dash diet and sodium reduction improve markers of bone turnover and calcium metabolism in adults. J. Nutr..

[37] Cashman K.D. (2002). Calcium intake, calcium bioavailability and bone health. Br. J. Nutr..

[38] Wilczynski C., Camacho P. (2014). Calcium use in the management of osteoporosis: Continuing questions and controversies. Curr. Osteoporos. Rep..

[39] Booth A., Camacho P. (2013). A closer look at calcium absorption and the benefits and risks of dietary *versus* supplemental calcium. Postgrad. Med..

[40] Benetou V., Orfanos P., Pettersson-Kymmer U., Bergstrom U., Svensson O., Johansson I., Berrino F., Tumino R., Borch K.B., Lund E. (2013). Mediterranean diet and incidence of hip fractures in a European cohort. Osteoporos. Intl..

