# Supplementary material for: Development of a Food Group-Based Diet Score and Its Association with Bone Mineral Density in the Elderly: The Rotterdam Study
Source: Nutrients. 2015 Aug 18;7(8):6974–90. doi: 10.3390/nu7085317 (PMC4555156; doi:10.3390/nu7085317)
Supplement: Supplementary File 1 [file nutrients-07-05317-s001.docx]

**Supplementary Materials**


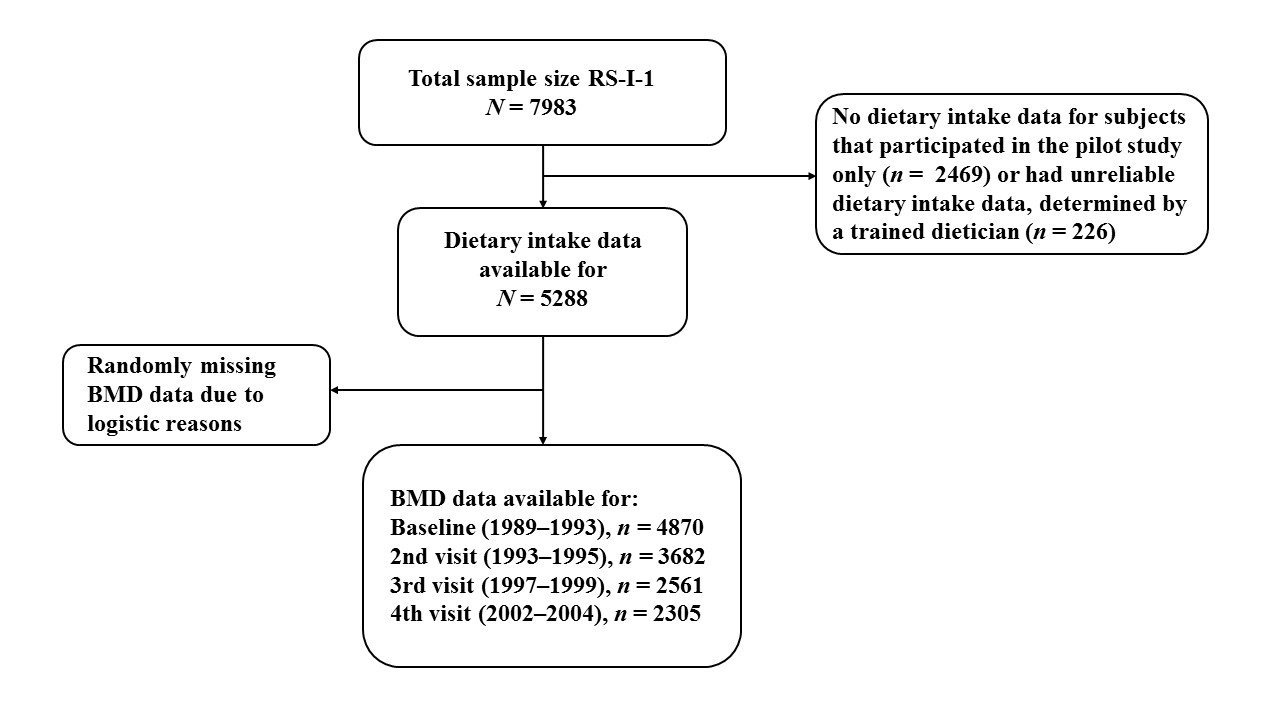


**Figure S1.** Sample sizes included for each measurement; RS-I-1: First cohort of the Rotterdam Study (details regarding the study design in Hofman *et al.*, 2013) [1]. BMD: bone mineral density.

**Table S1.** Details on the multiple imputation procedure.

| **Multiple Imputation Procedure** | | |
| --- | --- | --- |
| Software used | | SPSS 21 for windows. |
| Imputation method and key settings | | Fully conditional specification (Markov chain Monte Carlo method); Maximum iterations: 100. |
| Number of imputed data sets created | | 10 |
| Variable included in the imputation procedure and used in main analyses | Imputed and used as predictor | Body weight and height at all visits.  Physical activity (total time spend)  Lower limb disability index  Plasma Vitamin D  Highest education attained  Net household income  Smoking at baseline  Baseline prevalent type 2 diabetes  Use of lipid lowering and anti-hypertensive drugs  HRT use (females only) |
|  | Used as predictor only  (no missings or outcome variables) | BMD-Diet Score and Healthy diet Indicator (and all their components), alcohol intake, calcium intake, femoral neck BMD at all visits, baseline age, dietary supplement use |

**Table S1.** *Cont.*

| **Multiple Imputation Procedure** | |
| --- | --- |
| Software used | SPSS 21 for windows. |
| Variables not used in main analyses but used as predictors of missing data to increase plausibility of missing at random assumption | Other types of physical activity (time spend on medium  and vigorous activities), body mass index at every visit,  age at menopause |
| Treatment of non-normally  distributed variables | Predictive mean matching |
| Treatment of binary/categorical variables | Logistic regression |

BMD = bone mineral density; HRT = hormone replacement therapy.

**Table S2.** Values of imputed covariates before and after the multiple imputation process.

|  |  | **Original Data** | | **After Multiple Imputation** | |
| --- | --- | --- | --- | --- | --- |
| Height (cm) ^1^ |  |  | |  | |
| Baseline (1989–1993) |  | 167 | (161–174) | 166 | (161–174) |
|  | *Missing (n)* | *29* | |  | |
| 2nd visit (1993–1995) |  | 167 | (161–174) | 167 | (161–174) |
|  | *Missing (n)* | *113* | |  | |
| 3rd visit (1997–1999) |  | 166 | (160–173) | 166 | (160–173) |
|  | *Missing (n)* | *6* | |  | |
| 4th visit (2002–2004) |  | 165 | (160–173) | 166 | (160–173) |
|  | *Missing (n)* | *43* | |  | |
| Weight (kg) ^1^ |  |  | |  | |
| Baseline (1989–1993) |  | 73 | (66–81) | 73 | (66–81) |
|  | *Missing (n)* | *29* | |  | |
| 2nd visit (1993–1995) |  | 74 | (69–81) | 74 | (66–81) |
|  | *Missing (n)* | *114* | |  | |
| 3rd visit (1997–1999) |  | 74 | (65–82) | 74 | (66–82) |
|  | *Missing (n)* | *5* | |  | |
| 4th visit (2002–2004) |  | 75 | (66–84) | 75 | (67–74) |
|  | *Missing (n)* | *44* | |  | |
| Physical activity (h/day) ^1,2^ |  | 6.1 | (4.6–8.0) | 5.8 | (4.2–7.8) |
|  | *Missing (n)* | 19 | |  | |
| Plasma Vitamin D (nmol/L) ^1,3^ |  | 45 | (30–64) | 42 | (27–61) |
|  | *Missing (n)* | *192* | |  | |
| High education (%) |  | 37% | | 37% | |
|  | *Missing (n)* | *24* | |  | |
| High income (%) |  | 51% | | 52% | |
|  | *Missing (n)* | *489* | |  | |
| Smoking at baseline (%) |  | 23% | | 23% | |
|  | *Missing (n)* | *26* | |  | |
| Prevalent type 2 diabetes (%) |  | 10% | | 10% | |
|  | *Missing (n)* | *5* | |  | |

**Table S2.** *Cont.*

|  |  | **Original Data** | **After Multiple Imputation** |
| --- | --- | --- | --- |
| Use of lipid lowering agents (%) |  | 3% | 3% |
|  | *Missing (n)* | *4* |  |
| Use of anti-hypertensive drugs (%) |  | 13% | 13% |
|  | *Missing (n)* | *4* |  |
| Current or past HRT users (%) ^2^ |  | 9% | 9% |
|  | *Missing (n)* | *104* |  |

^1^: Median (interquartile range); ^2^: applicable to females only; ^3^: Physical activity and plasma Vitamin D were assessed at the 3rd visit (1997–1999) only. Main exposures (diet scores) and outcomes (BMD data) were not imputed; Abbreviations: BMD = bone mineral density; HRT = hormone replacement therapy.

**Table S3.** *A posteriori* defined dietary patterns that were shown to be associated with high BMD in the reviewed literature: Food groups and their factor loadings only food groups with factor loadings >0.3 or <−0.3 are displayed in this table.

| **Reference** | **Sample Size** | **Design  Dietary Data Assessment** | **Food Groups and Their Factor Loadings** | |
| --- | --- | --- | --- | --- |
|  |  |  | **Food Groups** | **Factor Loadings** |
| Shin, Br. J. Nutr., 2013 [2] | 3735 | Cross-sectional analysis, Single 24 h dietary recall | **“Dairy and fruit” pattern** | |
|  |  |  | Milk and dairy foods | 0.44 |
|  |  |  | Flour and bread | 0.44 |
|  |  |  | Fruits | 0.31 |
| Shin, Eur. J. Clin. Nutr., 2014 [3] | 1828 | Cross-sectional analysis in the Healthy Twin cohort  3-day food record | **“Fruit milk and whole grain” pattern** | |
|  |  |  | Fruits | 0.31 |
|  |  |  | **“Rice and kimichi” pattern** | |
|  |  |  | White rice | 0.35 |
|  |  |  | Kimichi | 0.32 |
| Langsetmo, BMC Musc. Disorders 2010 [4] | 6539 | Cohort study, the Canadian Multicentre Osteoporosis Study (CaMos),  self-administered FFQ | **“Nutrient dense” pattern** | |
|  |  |  | Vegetables | 0.56–0.61 *^1^ |
|  |  |  | Whole grains | 0.46 |
|  |  |  | Fish | 0.40 |
|  |  |  | Fruit | 0.48–0.52 *^1^ |
|  |  |  | Legumes | 0.37 |
| Kontogianni Nutrition,  2009 [5] | 220 | Cross-sectional design,  3-day food records | ***Pattern was not labeled*** | |
|  |  |  | Fish | 0.87 |
|  |  |  | Olive oil | 0.47 |
|  |  |  | Red meat | −0.31 |

**Table S3.** *Cont.*

| **Reference** | **Sample Size** | **Design  Dietary Data Assessment** | **Food Groups and Their Factor Loadings** | |  |
| --- | --- | --- | --- | --- | --- |
|  |  |  | **Food Groups** | **Factor Loadings** |  |
| Okubo, Am. J. Clin. Nutr.,  2006 [6] | 291 | Japanese Multi-centered Environmental  Toxicant Study (JMETS)  Cross-sectional  16-page self-administered diet history questionnaire (DHQ) to assess dietary habits in the previous month | **“Healthy” pattern** | |  |
|  |  |  | Fruit | 0.49 |  |
|  |  |  | (Processed) fish | 0.57/0.44 *^1^ |  |
|  |  |  | Vegetables (green/white/salted) | 0.61/0.40/0.30 |  |
|  |  |  | Mushrooms | 0.57 |  |
|  |  |  | Seaweeds | 0.36 |  |
|  |  |  | Soy products | 0.36 |  |
|  |  |  | Rice | −0.50 |  |
| Mc Naughton, J. Nutr. 2011 [7] | | 527 | Cross-sectional analysis  4-day food diary | **“Pattern 4” (not labeled)** | |
|  |  |  |  | Legumes | 0.54 |
|  |  |  |  | Seafood | 0.48 |
|  |  |  |  | Seeds and nuts | 0.47 |
|  |  |  |  | Wine | 0.38 |
|  |  |  |  | Rice (dishes) | 0.35 |
|  |  |  |  | Bacon and Ham | −0.32 |
| Mu, J. Am. Coll. Nutr., 2014 [8] | | 1319 | Cross-sectional analysis  FFQ on 19 food groups | **“Calcium food pattern”** | |
|  |  |  |  | Fresh fruit | 0.34 |
|  |  |  |  | Eggs | 0.64 |
|  |  |  |  | Marine products | 0.42 |
|  |  |  |  | Dairy products | 0.66 |
|  |  |  |  | Beans | 0.78 |
|  |  |  |  | Nuts | 0.39 |
|  |  |  |  | **“Traditional Chinese” pattern** | |
|  |  |  |  | Grains | 0.72 |
|  |  |  |  | Fresh vegetables | 0.76 |
|  |  |  |  | Fresh fruits | 0.60 |
|  |  |  |  | Pork | 0.51 |
| Whittle, Br. J. Nutr., 2012 [9]  *Also report the MD-Score* | | 489 | Cross-sectional analysis,  7-day diet history | **“Nuts and meat” pattern** *Young adult women only* | |
|  |  |  |  | Chips | 0.32 |
|  |  |  |  | Meat dishes | 0.37 |
|  |  |  |  | Red meat | 0.30 |
|  |  |  |  | Poultry | 0.34 |
|  |  |  |  | Chocolate | 0.53 |
|  |  |  |  | Confectionary | 0.34 |
|  |  |  |  | Crisps | 0.60 |
|  |  |  |  | Puddings | 0.31 |
|  |  |  |  | Condiments | 0.40 |

**Table S3.** *Cont.*

| **Reference** | **Sample Size** | **Design Dietary Data Assessment** | **Food Groups and Their Factor Loadings** | |
| --- | --- | --- | --- | --- |
|  |  |  | **Food Groups** | **Factor Loadings** |
| De Jonge, 2015 (submitted work) [10] | 5435 | Longitudinal analysis,  FFQ on 172 food items | **“Mediterranean**-**like”**-**pattern** |  |
|  |  |  | Poultry | 0.49 |
|  |  |  | Fatty fish | 0.52 |
|  |  |  | Lean and battered fish | 0.63 |
|  |  |  | Shell fish | 0.33 |
| Tucker, Am. J. Clin Nutr. [11] | 907 | Cross-sectional analysis,  Framingham Heart Study  FFQ on 162 food items | **“Fruit, vegetables and cereal” group** *Cluster analysis: factor loadings not applicable* | |
|  |  |  | High in:  Citrus fruit and juice, other fruit and juice  dark-green vegetables, other vegetables  whole milk, breakfast cereals  Low in:  Red and processed meat, liquor, beer, candy and soft drinks | |

*^1^: If specific food groups were divided into subgroups (e.g., white, yellow or leafy vegetables), a range of the corresponding factor loadings is provided; BMD = bone mineral density; FFQ = food frequency questionnaire;
*MD-Score: Mediterranean Diet Score.*

**Table S4.** *A posteriori* defined dietary patterns that were shown to be associated with **low** BMD in the reviewed literature: Food groups and their factor loadings; Only food groups with factor loadings >0.3 or <−0.3 are displayed in this table.

| **Reference** | **Sample Size** | **Design  Dietary Data Assessment** | **Food Groups and Their Factor Loadings** | |
| --- | --- | --- | --- | --- |
|  |  |  | **Food Groups** | **Factor Loadings** |
| Shin, Br. J. Nutr., 2013 [2] | 3735 | Cross-sectional analysis,  Single 24 h dietary recall | **“White rice, kimichi and** **seaweed” pattern** | |
|  |  |  | White rice | 0.34 |
|  |  |  | Seaweed | 0.37 |
|  |  |  | Kimichi | 0.32 |
|  |  |  | Noodles & dumplings | −0.34 |

**Table S4.** *Cont.*

| **Reference** | **Sample Size** | **Design  Dietary Data Assessment** | **Food Groups and Their Factor Loadings** | |
| --- | --- | --- | --- | --- |
|  |  |  | **Food Groups** | **Factor Loadings** |
| Karamati, Calc. Tissue Int., 2012 [12] | 160 | Cross-sectional analysis,  168-item FFQ developed for the Tehran Lipid and Glucose Study | **“High in SFA” pattern** | |
|  |  |  | High fat dairy | 0.79 |
|  |  |  | Organ meat | 0.74 |
|  |  |  | Red/Processed meats | 0.49 |
|  |  |  | Non- refined cereals | 0.43 |
|  |  |  | Eggs | 0.40 |
|  |  |  | Low fat dairy | −0.32 |
|  |  |  | **“High in processed foods” pattern** | |
|  |  |  | French fries | 0.70 |
|  |  |  | Mayonnaise | 0.69 |
|  |  |  | Sweets and desserts | 0.56 |
|  |  |  | Vegetable oils | 0.42 |
|  |  |  | Legumes | 0.47 |
|  |  |  | Refined cereals | 0.32 |
|  |  |  | Fish | −0.30 |
| Langsetmo, BMC Musc. Disorders 2010 [4] | 6539 | Cohort study, the Canadian Multicentre Osteoporosis Study, self-administered FFQ | **“Energy dense” pattern** | |
|  |  |  | Soft drinks | 0.42 |
|  |  |  | Processed meat products | 0.50–0.56 |
|  |  |  | Sweets | 0.47 |
|  |  |  | High fat potatoes | 0.58 |
| Okubo, Am. J. Clin. Nutr., 2006 [6] | 291 | Japanese Multi-centered Environmental Toxicant Study  Cross-sectional  16-page self-administered diet history questionnaire to assess dietary habits in the previous month | **“Western pattern”** | |
|  |  |  | White vegetables | 0.33 |
|  |  |  | Fats and oils | 0.62 |
|  |  |  | Meat | 0.59 |
|  |  |  | Processed Meats | 0.54 |
|  |  |  | Butter | 0.41 |
|  |  |  | Seasonings | 0.37 |
|  |  |  | Soups | 0.30 |
|  |  |  | Rice | −0.28 |
|  |  |  | Sweets | −0.42 |
|  |  |  | Salted vegetables | −0.37 |
|  |  |  | Meat | 0.34 |
| Hardcastle, Eur. J. Clin. Nutr., 2011 [13] | 3236 | Cross-sectional analysis in the Aberdeen Prospective Osteoporosis Study FFQ of 98 foods | **‘Processed food pattern’** | |
|  |  |  | Pulse | 0.30 |
|  |  |  | Cake | 0.39 |
|  |  |  | Cereal | 0.30 |
|  |  |  | Dessert | 0.37 |
|  |  |  | Dried/tinned fruit | 0.32 |
|  |  |  | Soup | 0.34 |
|  |  |  | Bread | −0.57 |
|  |  |  | Fats/oils | −0.59 |

**Table S4.** *Cont.*

| **Reference** | **Sample Size** | **Design  Dietary Data Assessment** | **Food Groups and Their Factor Loadings** | | |
| --- | --- | --- | --- | --- | --- |
|  |  |  | **Food Groups** | | **Factor Loadings** |
| Whittle, Br. J. Nutr., 2012 [9]  *Also report the  MD-Score* | 489 | Cross-sectional analysis,  7-day diet history | **“Refined” pattern** *Young adult men only* | | |
|  |  |  | Chips | | 0.53 |
|  |  |  | Soft drinks | | 0.45 |
|  |  |  | Chocolate | | 0.51 |
|  |  |  | Confectionary | | 0.48 |
|  |  |  | Crisps | | 0.44 |
|  |  |  | Puddings | | 0.54 |
|  |  |  | Condiments | | 0.52 |
|  |  |  | Hot drinks | | −0.35 |
| De Jonge, 2015 (submitted work) [10] | 5435 | Longitudinal analysis,  FFQ 172 on food items | **“Processed Food” pattern** | | |
|  |  |  | Processed Meat | | 0.45 |
|  |  |  | Alcoholic drinks | | 0.56 |
|  |  |  | Mixed meals | | 0.37 |
|  |  |  | Fruit | | −0.55 |
|  |  |  | Yoghurt | | −0.51 |
| Fairweather-Tait, Am. J. Clin. Nutr. 2011 [14,15] | 2464 | Cross-sectional analysis,  FFQ on 131 items | **“Traditional 20th-century  English” pattern** | | |
|  |  |  | Meat | 0.34 | |
| Tucker, Am. J. Clin. Nutr. 2014 [11] | 907 | Cross-sectional analysis,  Framingham Heart Study  FFQ on 162 food items | **“Candy group”** *Cluster analysis: factor loading not applicable* | | |
|  |  |  | High in: Candy  Low in: Citrus fruit and juice, dark-green vegetables, other vegetables, fish | | |

BMD = bone mineral density; FFQ = food frequency questionnaire; *MD-Score: Mediterranean Diet Score.*

**Table S5.** Details on *a priori* defined Diet Scores that were shown to be associated with high BMD.

| **Reference** | **Name of the Score** | **Scores Associated with High BMD** | | **Other Aspects of the Score** | **Reflects** |
| --- | --- | --- | --- | --- | --- |
|  |  | **High in** | **Low in** |  |  |
| Bhupathiraju,  Am. J. Clin. Nutr. 2013 [15] | Diet and Lifestyle Score of the American Heart Association | Fruits and vegetables  Whole grain, high fibre foods  (Oily) fish | SAFA, trans fat and cholesterol  Beverages and foods with added sugar  Salty foods  Alcohol (in moderation) | Balance kcal intake and physical activity | 2006 Diet and Lifestyle recommendations of the American Heart Association for CVD risk reduction |
| Whittle, Br. J. Nutr. 2012 [9] | Dietary Diversity Score  (counted servings 1 to 5)– 30 g of solid foods and 60 g of liquids needed to be consumed | Dairy, meat, grain,  fruit and vegetable | - | - | Diversity of the diet |
| Rivas, Int. J. Food. Sci. Nutr. [16] | Mediterranean Diet Score | Vegetables, legumes,  fruit and nuts, cereal, fish | Lipid ratio—ratio of monounsaturated fat to saturated fat | - | Adherence to a Mediterranean diet |
| Go, Nutr. Res. Pract., 2014 [17] | Dietary Diversity Score  Food group intake patterns | Dairy, meat, grain,  fruit and vegetable | - | - | Diversity of the diet |

Abbreviations: BMD = bone mineral density; CVD = cardiovascular disease; SAFA = Saturated fatty aciss; -: not applicable.

**Table S6.** Food groups and corresponding food items included in the Score.

|  | **Food Groups** | **Summary of Food Items Included** |
| --- | --- | --- |
| **“High-BMD”  components** | 1. **Vegetables** | All vegetables raw or boiled, gherkins,  vegetable juices, mushrooms |
|  | 1. **Fruits** | Fresh fruits, dried fruits, fruit cocktail in syrup, fruit juices |
|  | 1. **Dairy** | Skimmed, semi- skimmed and full fat milk, buttermilk, low fat yoghurt with and without fruits, low fat and half fat fromage frais |
|  | 1. **Whole grain products** | Whole grain bread, pasta, muesli, rye bread |
|  | 1. **Fish** | Cod, Fish fingers, Fish lean 0–2 g fat raw, Haddock fillet in batter fried, Eel, Fish, 2–10 g fat and > 10 g fat raw, Herring, Mackerel, Plaice Salmon Sardines/pilchards (fresh and canned), mussels, shrimps |
|  | 1. **Legumes and beans** | Beans, peas, chickpeas |
| **“Low-BMD”  components** | 1. **Meat (red, processed, organ)** | Bacon, Beef salted and smoke dried, Corned beef, Croquette meat ragout deep fat fried, ham, sausages and salami, Beef raw, Hamburger, Horsemeat, Lamb, Liver chicken/ox/pork, Mutton, Pork, Veal |
|  | 1. **Confectionary** | Pie, biscuits, cake, chocolate bars, spiced honey cake, gateau, honey, popsicle ice cream, candy, pancakes, praline, sugar and sweet bread toppings |

BMD: bone mineral density.

**Table S7.** Median dietary intake (in g/day) of each food group that is included in the
BMD-Diet Score.

| **“High BMD” Food Groups** | **Ascending Values Based on Quartiles** | | | |
| --- | --- | --- | --- | --- |
|  | **1** | **2** | **3** | **4** |
| Vegetables | 122 | 210 | 295 | 425 |
| Fruits | 109 | 161 | 207 | 281 |
| Dairy products | 164 | 318 | 453 | 674 |
| Whole grain products | 45 | 101 | 135 | 185 |
| Fish | 0 | 7 | 16 | 36 |
| Legumes and beans ***** | **Ascending values based on the median *** | | | |
|  | **1** | | **2** | |
|  | 0 | | 11 | |
| **“Low BMD” food groups** | **Descending values based on quartiles** | | | |
|  | **4** | **3** | **2** | **1** |
| Meat | 48 | 80 | 105 | 146 |
| Confectionary | 27 | 57 | 88 | 135 |

*****: For the food group “legumes and beans” scores were based on the median rather than quartiles as the distribution of intake of this food group did not allow computation of quartiles; BMD: bone mineral density.

**Table S8.** Components of our BMD-Diet Score *versus* those of the DASH-diet.

|  | | **BMD**-**Diet Score  *Newly Developed*** | **DASH**-**Diet  (Lin, J. Nutr., 2003)** |
| --- | --- | --- | --- |
| **Favorable** | **Food groups** | Vegetables | Vegetables |
|  |  | Fruits | Fruits |
|  |  | Fish | Fish |
|  |  | Dairy Whole grains | Low fat dairy Whole grains |
|  |  | Legumes and beans | |
|  |  |  | Poultry |
|  | **Nutrients** | *Not applicable* | *Not applicable* |
| **Unfavorable** |  | Meat | Red meat |
|  | **Food groups** | Confectionary | Sweets |
|  |  |  | Sugar containing beverages |
|  | **Nutrients** |  | Fats |

Abbreviations: BMD = Bone Mineral Density, DASH = Dietary Approach to Stop Hypertension; In this table, the label “favorable” corresponds to the High-BMD components and “unfavorable” to the Low-BMD components of the BMD-Diet Score.

Reference

1. Hofman, A.; Murad, S.D.; van Duijn, C.M.; Franco, O.H.; Goedegebure, A.; Ikram, M.A.;
   Klaver, C.C.W.; Nijsten, T.E.C.; Peeters, R.P.; Stricker, B.H.C.; *et al.* The Rotterdam Study: 2014 objectives and design update. *Eur. J. Epidemiol.* **2013**, *28*, 889–926.
2. Shin, S.; Joung, H. A dairy and fruit dietary pattern is associated with a reduced likelihood of osteoporosis in Korean postmenopausal women. *Br. J. Nutr.* **2013**, *110*, 1926–1933.
3. Shin, S.; Sung, J.; Joung, H. A fruit, milk and whole grain dietary pattern is positively associated with bone mineral density in Korean healthy adults. *Eur. J. Clin. Nutr.* **2014**, *69*, 442–428.
4. Langsetmo, L.; Poliquin, S.; Hanley, D.A.; Prior, J.C.; Barr, S.; Anastassiades, T.; Towheed, T.; Goltzman, D.; Kreiger, N. Dietary patterns in Canadian men and women ages 25 and older: Relationship to demographics, body mass index, and bone mineral density. *BMC
   Musculoskelet. Disord.* **2010**, *11*, 20, doi:10.1186/1471-2474-11-20.
5. Kontogianni, M.D.; Melistas, L.; Yannakoulia, M.; Malagaris, I.; Panagiotakos, D.B.; Yiannakouris, N. Association between dietary patterns and indices of bone mass in a sample of Mediterranean women. *Nutrition* **2009**, *25*, 165–171.
6. Okubo, H.; Sasaki, S.; Horiguchi, H.; Oguma, E.; Miyamoto, K.; Hosoi, Y.; Kim, M.K.; Kayama, F. Dietary patterns associated with bone mineral density in premenopausal Japanese farmwomen.
   *Am. J. Clin. Nutr.* **2006**, *83*, 1185–1192.
7. McNaughton, S.A.; Wattanapenpaiboon, N.; Wark, J.D.; Nowson, C.A. An energy-dense, nutrient-poor dietary pattern is inversely associated with bone health in women. *J. Nutr.* **2011**, *141*, 1516–1523.
8. Mu, M.; Wang, S.F.; Sheng, J.; Zhao, Y.; Wang, G.X.; Liu, K.Y.; Hu, C.L.; Tao, F.B.; Wang, H.L. Dietary patterns are associated with body mass index and bone mineral density in Chinese freshmen. *J. Am. Coll. Nutr.* **2014**, *33*, 120–128.
9. Whittle, C.R.; Woodside, J.V.; Cardwell, C.R.; McCourt, H.J.; Young, I.S.; Murray, L.J.;
   Boreham, C.A.; Gallagher, A.M.; Neville, C.E.; McKinley, M.C. Dietary patterns and bone mineral status in young adults: The Northern Ireland young hearts project. *Br. J. Nutr.* **2012**, *108*, 1494–1504.
10. De Jonge, E.A.L.; Rivadeneira, F.; Erler, N.S.; Hofman, A.; Uitterlinden, A.G.; Franco, O.H.; Kiefte-de Jong, J.C. Dietary patterns in an elderly population and their relation with bone mineral density: The Rotterdam Study, 2015, submitted for publication.
11. Tucker, K.L.; Chen, H.; Hannan, M.T.; Cupples, L.A.; Wilson, P.W.; Felson, D.; Kiel, D.P. Bone mineral density and dietary patterns in older adults: The Framingham Osteoporosis Study. *Am. J. Clin. Nutr.* **2002**, *76*, 245–252.
12. Karamati, M.; Jessri, M.; Shariati-Bafghi, S.E.; Rashidkhani, B. Dietary patterns in relation to bone mineral density among menopausal iranian women. *Calcif. Tissue Intl.* **2012**, *91*, 40–49.
13. Hardcastle, A.C.; Aucott, L.; Fraser, W.D.; Reid, D.M.; Macdonald, H.M. Dietary patterns, bone resorption and bone mineral density in early post-menopausal scottish women. *Eur. J. Clin. Nutr.* **2011**, *65*, 378–385.
14. Fairweather-Tait, S.J.; Skinner, J.; Guile, G.R.; Cassidy, A.; Spector, T.D.; MacGregor, A.J. Diet and bone mineral density study in postmenopausal women from the twinsuk registry shows a negative association with a traditional English dietary pattern and a positive association with wine. *Am. J. Clin. Nutr.* **2011**, *94*, 1371–1375.
15. Bhupathiraju, S.N.; Lichtenstein, A.H.; Dawson-Hughes, B.; Hannan, M.T.; Tucker, K.L. Adherence to the 2006 American heart association diet and lifestyle recommendations for cardiovascular disease risk reduction is associated with bone health in older Puerto Ricans. *Am. J. Clin. Nutr.* **2013**, *98*, 1309–1316.
16. Rivas, A.; Romero, A.; Mariscal-Arcas, M.; Monteagudo, C.; Feriche, B.; Lorenzo, M.L.; Olea, F. Mediterranean diet and bone mineral density in two age groups of women. *Intl. J. Food Sci. Nutr.* **2013**, *64*, 155–161.
17. Go, G.; Tserendejid, Z.; Lim, Y.; Jung, S.; Min, Y.; Park, H. The association of dietary quality and food group intake patterns with bone health status among Korean postmenopausal women: A study using the 2010 Korean national health and nutrition examination survey data. *Nutr. Res. Pract.* **2014**, *8*, 662–669.

© 2015 by the authors; licensee MDPI, Basel, Switzerland. This article is an open access article distributed under the terms and conditions of the Creative Commons Attribution license (http://creativecommons.org/licenses/by/4.0/).
